# Supplementary material for: Three-Dimensional Bioprinting of Organoid-Based Scaffolds (OBST) for Long-Term Nanoparticle Toxicology Investigation
Source: Int J Mol Sci. 2023 Apr 1;24(7):6595. doi: 10.3390/ijms24076595 (PMC10095512; doi:10.3390/ijms24076595)
Supplement: Supplementary file 1 [file ijms-24-06595-s001.zip › ijms-2291145-supplementary.pdf]

Supplementary Table S1. Cell cycle in unprinted and printed cells at different time-ponts. Mean±SD. N=3 each.

	2D	3D T0	3D T24h	3D T72h
G0/G1	63.7±3.6	65.2±3.8	64.8±3.9	80.6±4.2
S	18.2±1.8	14.7±1.3	14.8±1.6	10.5±1.2
G2/M	18.1±1.9	20.1±1.9	20.4±2.1	8.9±1.1

A

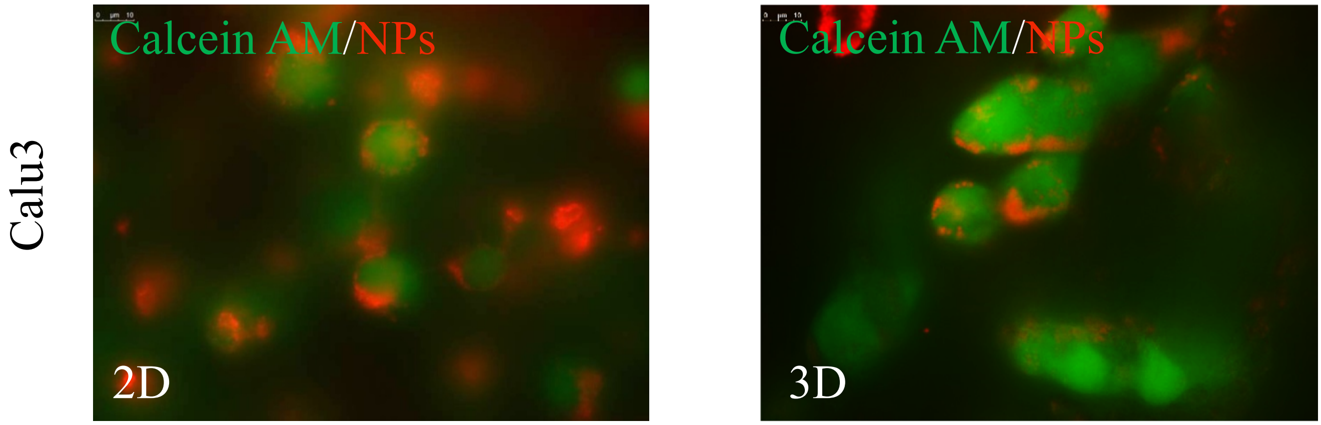

Supplementary Figure S1. Live death assay for Calu-3 cells in the presence of fluorescent non-toxic carboxyl-modified 40 nm nanoparticles internalization. Left: conventional 2D culture. Right: 3D Printed OBST

Supplementary Figure S1
